# Validation of Thigh Angle Estimation Using Inertial Measurement Unit Data against Optical Motion Capture Systems

**DOI:** 10.3390/s19030596

**Published:** 2019-01-31

**Authors:** Nimsiri Abhayasinghe, Iain Murray, Shiva Sharif Bidabadi

**Affiliations:** 1 Department of Electrical and Computer Engineering, Sri Lanka Institute of Information Technology, Malabe 10115, Sri Lanka; 2 School of Electrical Engineering, Computing and Mathematical Sciences, Curtin University of Technology, Perth 6102, Australia; I.Murray@curtin.edu.au; 3 School of Civil and Mechanical Engineering, Curtin University of Technology, Perth 6102, Australia; Shiva.Sharif@curtin.edu.au

**Keywords:** human gait analysis, inertial measurement units, sensor fusion

## Abstract

Inertial measurement units are commonly used to estimate the orientation of sections of sections of human body in inertial navigation systems. Most of the algorithms used for orientation estimation are computationally expensive and it is difficult to implement them in real-time embedded systems with restricted capabilities. This paper discusses a computationally inexpensive orientation estimation algorithm (Gyro Integration-Based Orientation Filter—GIOF) that is used to estimate the forward and backward swing angle of the thigh (thigh angle) for a vision impaired navigation aid. The algorithm fuses the accelerometer and gyroscope readings to derive the single dimension orientation in such a way that the orientation is corrected using the accelerometer reading when it reads gravity only or otherwise integrate the gyro reading to estimate the orientation. This strategy was used to reduce the drift caused by the gyro integration. The thigh angle estimated by GIOF was compared against the Vicon Optical Motion Capture System and reported a mean correlation of 99.58% for 374 walking trials with a standard deviation of 0.34%. The Root Mean Square Error (RMSE) of the thigh angle estimated by GIOF compared with Vicon measurement was 1.8477°. The computation time on an 8-bit microcontroller running at 8 MHz for GIOF is about a half of that of Complementary Filter implementation. Although GIOF was only implemented and tested for estimating pitch of the IMU, it can be easily extended into 2D to estimate both pitch and roll.

## 1. Introduction

Inertial measurement units (IMU) are often used to estimate orientation and motion of human body or limbs in human positioning and navigation systems. Most of these systems use trunk [1] or foot [2] movement to identify human gait and they usually use relatively computationally expensive orientation estimation algorithms often utilizing Kalman filtering [3,4,5].

Although Kalman filters are fast enough in personal computers or workstations, implementing them in low-end real-time embedded systems is not an easy task. One main drawback in using these techniques is that the embedded devices have to be capable of executing these computationally expensive algorithms in real-time, usually a 32-bit microcontroller with a digital signal processor (DSP), which increases the financial cost. The work discussed in this paper is a part of a project that develops a navigation aid for vision impaired people to assist navigating in unfamiliar environments. Hence, one of the main aims was to reduce the computational complexity of algorithms and thereby reduce the financial cost of the devices.

**Figure 1:**
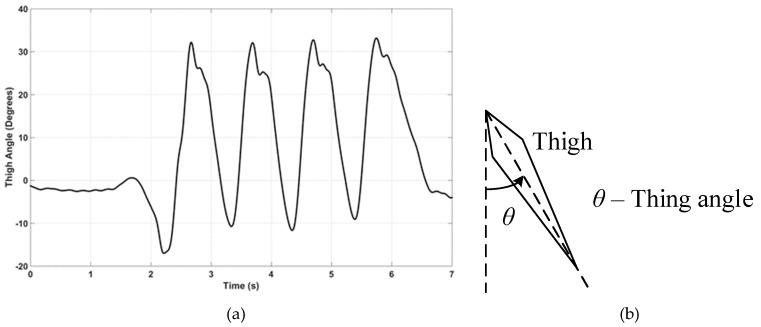
(**a**) Thigh angle of a subject during a single trial measured from the motion analysis lab (MAL). (**b**) Thigh angle measuring reference.

This paper discusses a computationally economical algorithm (Gyro Integration-Based Orientation Filter—GIOF), that may be implemented in a low-cost 8-bit microcontroller, to estimate single dimensional orientation by fusing accelerometer and gyroscopic data of an IMU. GIOF is used to estimate the forward and backward swing angle of the thigh (herein after referred to as the “thigh angle”), as illustrated in Figure 1b, taking forward swing as positive. The thigh angle calculated using GIOF is validated against Vicon Optical Motion Analysis system, which is well documented to be accurate enough for measuring motion of human body for clinical and rehabilitation purposes [6].

## 2. Materials and Methods

The main concern of the vision impaired navigation system was the financial cost which drove us to find less computationally expensive techniques for pedestrian tracking. Further, the positioning of the sensors has to be convenient for vision impaired people as they may have difficulty with accurate placement of said devices.

It was observed that the trouser pocket and shirt pocket are common carrying positions of mobile phones for males whereas the trouser pocket and the hand bag are common carrying positions for females. Based on the experimental results discussed in [7], it was observed that the trouser pocket is a better position to keep the tracking device so that the thigh movement, which directly relates to human gait, can easily be tracked. It was also observed that the gyroscope signal alone provides sufficient information to track the flexion and extension movement of the thigh during the stride cycle. Further, it was empirically proven in [8] that a gyroscope-based thigh-mounted pedometer gives close to 100% accuracies and that Kwon et al. have used that algorithm in their cross-platform pedometer system and reported errors less than 1% in most cases [9].

**Figure 2:**
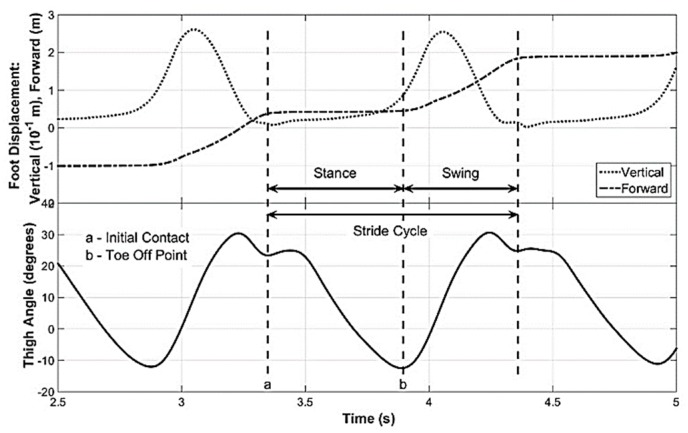
Thigh angle synchronized with the vertical and forward movements of the foot. Vertical and forward movements of the foot is used to identify the key points of the thigh angle waveform.

A sample of the flexion–extension movement of the thigh (thigh angle) when the subject is walking on a level surface is shown in Figure 1a. This thigh angle was obtained from an experiment conducted in a Motion Analysis Lab (MAL) containing a Vicon [6] optical motion capture system, which is discussed later. To enable identifying key points of stride cycle in the thigh angle waveform, the foot movement was also recorded. Figure 2 shows a time synchronized plot of the thigh angle with the vertical and forward movements of the foot. By observing the foot movement waveforms, it can be seen that the local minimum (point ‘a’ of Figure 2) of the thigh angle waveform next to the maximum refers to the Initial Contact point of the stride cycle. Further, it can be seen that the minimum (point ‘b’ of Figure 2) of the thigh angle waveform is the toe-off point of the stride cycle, which can be detected by minima detection. Toe-off may also be identified by detecting the positive zero crossing of the gyro signal, which refers to the minimum of the thigh angle waveform. Gait phase detection based on thigh-mounted IMU data is discussed in the discussion of this paper that may be considered in future work. The stride cycle and its two main phases—stance and swing—are also marked in the Figure 2.

**Figure 3:**
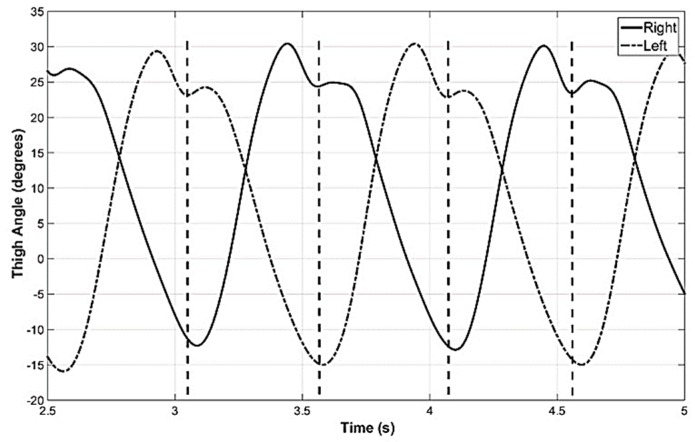
Thigh angle of left and right legs. This indicates that the toe off of one leg is synchronized with the initial contact of the other leg as shown by vertical dashed lines.

The synchronization of the movement of the two thighs was also measured and depicted in Figure 3. It can be seen that the toe-off of one leg is closely synchronized to the initial contact of the other leg. These observations indicate that proper estimation of the thigh angle of one leg is sufficient to identify key points of the stride cycle of both legs of subjects without gait impairment. However, these observations were made with the Vicon optical motion capture system and it is necessary to compare the thigh angle computed with GIOF for the purpose of validation.

The validation experiment was conducted with the participation of 9 female and 10 male volunteer participants. All participants are non-vision impaired and known to have no other impairment or disability. The experimental setup, the procedures followed in the experiment and the analysis techniques are discussed in this sections. Data collected from the Vicon system in the motion analysis lab (MAL) and IMU data were recorded in two independent systems. How they were synchronized in the analysis is also discussed in this section.

**Figure 4:**
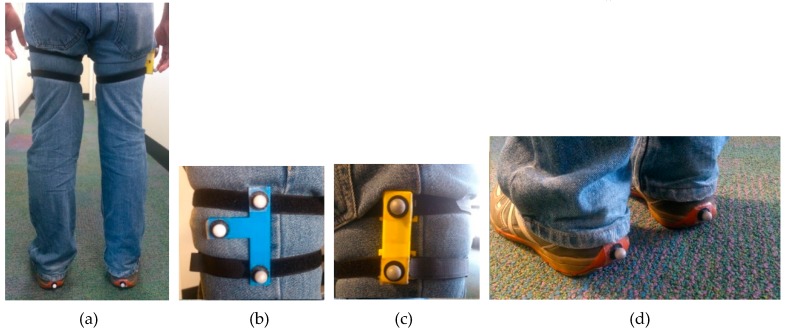
Marker and IMU placement. (**a**) Full placement. (**b**) Marker cluster attached to the left thigh of subject and the markers on it. (**c**) Custom-made IMU attached on the right thigh and the markers placed on it. (**d**) Markers attached to the heels.

The experiment was conducted in the motion analysis lab of the School of Physiotherapy and Exercise Science of Curtin University, so that the results of the new orientation estimation algorithm (GIOF) can be compared with the angles estimated by the optical motion capture system. The MAL has a Vicon optical motion capture system with 14 semi-infrared cameras. Retro-reflective spherical markers were used to capture the motion of the thighs and the feet of the subjects. Figure 4 shows the marker positioning on the subject’s legs. A custom-made IMU built using MPU-9150, as discussed in [10], was attached to the right thigh of the subject and two markers were placed on that as shown in Figure 4c to capture the tilt of the IMU. A marker cluster was attached to the subject’s left thigh to capture the thigh angle of the left leg. A marker was attached to the heel of each shoe to capture the movement of the heel (or the foot), so that the Initial Contact and the toe-off points can be identified.

The Vicon system was calibrated before collecting data of each batch of test subjects, so that the reading is accurate enough as a reference [11]. The IMU data were recorded in a laptop that operates independent of the Vicon system. Both data were sampled at 100 samples per second. IMU and Vicon data logging were manually triggered separately before the subject started their trial. The subjects were asked to walk in a straight line with their natural walking style with normal gait of ~2 steps per second. Both MAL and IMU data were recorded for 20 walking trials (10 trials in each direction of the MAL capture area) per each subject.

As MAL and IMU data were recorded in independent systems, they were preprocessed separately. Vicon Nexus software was used to preprocess MAL data and export coordinates of each marker into comma separated value (CSV) format. These coordinates were then used to compute the thigh angles. MATLAB was used to perform these calculations and all other calculations discussed in this paper.

The algorithm discussed below was used to compute the thigh angle using IMU data. Both angle data were then resampled (linearly interpolated) at 1000 samples per second and sent through an extrema detection algorithm to pick all maxima and minima of the two waveforms. Both waveforms were then trimmed at identical points of the two waveforms starting from a minimum and ending at a minimum. The identical points were identified manually by comparing the two waveforms. These trimmed waveforms were then used to compute the correlation coefficients and the error characteristics between the two methods.

The thigh angle is estimated by fusing accelerometer and gyroscope data. The gyroscopic signal is low-pass-filtered using a moving average filter with a 10-sample window size to remove the high frequency noise. Further, another moving average low-pass filter was used to compute the static error of the gyroscope and remove it from the gyroscopic signal. When compared with the thigh angle computed from MAL data, it was observed that the gyro integration does not drift when the window size is 150 samples. One-hundred-and-fifty samples are approximately two strides long. The sample being considered was placed at the center of the window of the moving average filter in both cases.

**Figure 5:**
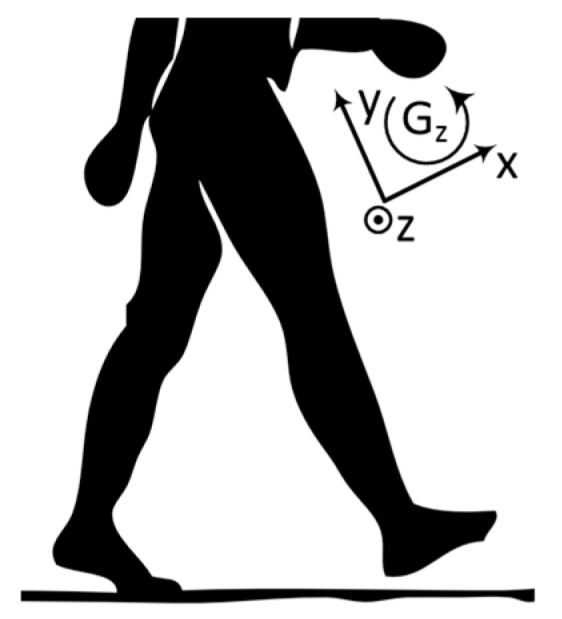
Reference axis of IMU data.

The reference axis of the IMU is shown in Figure 5 and it is clear that only gyroscopic z axis data is needed to measure the angular velocity of forward and backward movements of the thigh.

The thigh angle estimation algorithm discussed in this paper is an improved version of the algorithm discussed in [12]. Similar technique to the algorithm discussed in [12] is used in [13] by Diaz et al., and both contained an incorrect assumption. They perform the zero acceleration update when the total acceleration measured by the accelerometer sensor is less than a certain threshold (typically gravitational acceleration + noise margin). However, it was noted that the total acceleration can be less than gravity when the IMU is in acceleration against gravity. Hence, one condition for zero acceleration update was taken as

(1) $$\gamma_{1}<\sqrt{a_{x_{k}}^{2}+a_{y_{k}}^{2}+a_{z_{k}}^{2}}<\gamma_{2}$$

where $a_{i_{k}}$ (i=x,y,z) is the acceleration of i axis measured at time stamp *k*. The parameters $\gamma_{1}$ and $\gamma_{2}$ are selected to accommodate the noise embedded in the accelerometer reading. $\gamma_{1}$ and $\gamma_{2}$ were selected such that $\gamma_{1}=g-\eta$ and $\gamma_{2}=g+\eta$ where g is gravitational acceleration and η is the noise margin allowed.

The second condition for taking zero acceleration updates is the angular velocity to be close to zero, which implies that the thigh is not in movement. It was observed that this condition improves the smoothness and the accuracy of the thigh angle. The condition is given as

(2) $$g_{k_{z}}<\gamma_{3}$$

where $g_{k_{z}}$ is the angular velocity of z axis measured at time stamp *k* and $\gamma_{3}$ is selected to accommodate error in gyro data.

**Figure 6:**
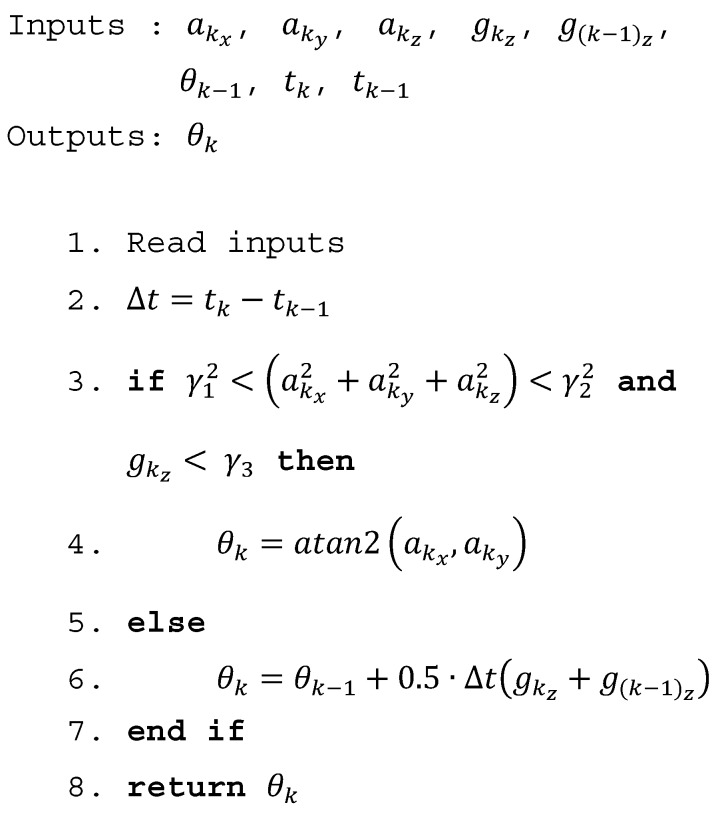
Gyro Integration-Based Orientation Filter (GIOF). $a_{i_{k}}$ (i=x,y,z) is the acceleration of i axis measured at time stamp *k*; $g_{k_{z}}$ and $g_{{\left(k-1\right)}_{z}}$ are gyroscope z axis reading measured at time stamps *k* and (*k* − 1); $t_{k}$ and $t_{k-1}$ are the time values of time stamps *k*; and (*k* − 1); $\theta_{k-1}$ is the previous estimation of thigh angle and $\theta_{k}$ is the current estimation of thigh angle.

When the conditions in (1) and (2) are satisfied, thigh angle update is taken from the accelerometer reading and otherwise integrates the gyroscope to get the thigh angle update. Trapezoidal rule was used in the integration of the gyro reading instead of using rectangle method, which is commonly used in literature. Trapezoidal rule was used because that gives a better estimation than the rectangle method. The GIOF is shown as pseudocode in Figure 6. Note that the square root calculation of (1) is not performed in the algorithm and $\gamma_{1}$ and $\gamma_{2}$ are squared instead, to reduce the computational demand.

The research presented in this paper was conducted in accordance with the National Health and Medical Research Council National Statement on Ethical Conduct in Human Research (2007)–updated March 2014. The presented research study received human research ethics approval from the Curtin University Human Research Ethics Committee (EC00262), Approval Number SMEC-105-11 and PT263/2013.

## 3. Results

### 3.1. Validation of GIOF Against MAL

**Figure 7:**
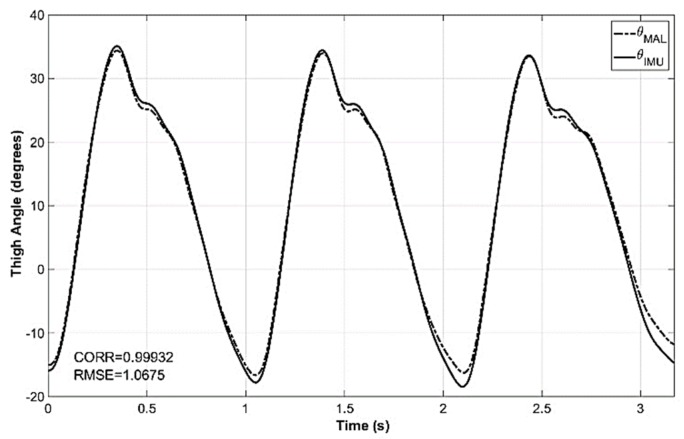
Thigh angle computed from MAL data $\left(\theta_{MAL}\right)$ and IMU data $\left(\theta_{IMU}\right)$ for a sample trial. Correlation coefficient of the two waveforms is 0.99932 and the root mean square error (RMSE) is 1.0675 for this trial.

Trimmed versions of the thigh angle computed using MAL data ($\theta_{MAL}$) and IMU data ($\theta_{IMU}$) were used to compute the correlation of the two waveforms. $\theta_{MAL}$ and $\theta_{IMU}$ of one of the trials are shown in Figure 7 with the correlation coefficient. It was observed that the correlation of the two waveforms was higher when the correlation was calculated for a single stride cycle. However, the intention was to find the correlation for all the strides of the trial. The analysis of 361 trials of 19 subjects (10 male and nine female) reported a mean correlation of 99.58% between $\theta_{IMU}$ and $\theta_{MAL}$ with a standard deviation of 0.34%. The maximum and minimum correlations reported were 99.96% and 97.33%, respectively. The correlation statistics of the trials are shown in Table 1.

**Figure 8:**
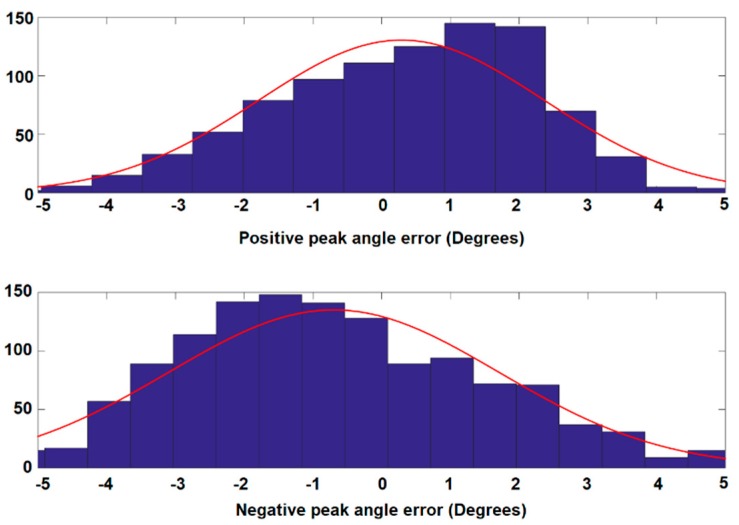
Histograms of positive and negative peak angle errors.

In addition to estimating correlation of the two waveforms, the error of the peak values was also calculated to check the goodness of the thigh angle estimation. The error of the estimated peak value was considered because the maximum and the minimum of the thigh angle are used to estimate the open angle of the stride. The histograms of the positive peak error and negative peak error are shown in Figure 8. The distributions show that the majority of error lies between ±3°. However, the peak of the error distribution of positive peak angle is shifted in the positive direction and that of the negative peak angle is shifted in the negative direction. This implies that there is a scaling error between the MAL measurement and the estimation from GIOF. The RMSE for the positive and negative peaks are 2.0954° and 2.4967°, respectively. The RMSE of each trial was also calculated and the mean of RMSE for all 374 trials was 1.8477° with a standard deviation of 0.56766°.

**Figure 9:**
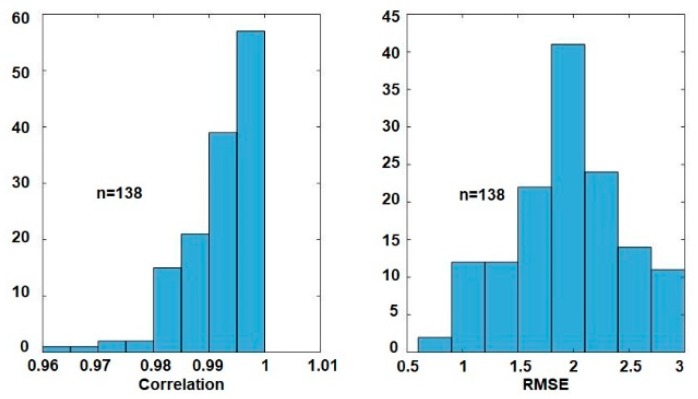
Distributions of correlation and RMSE between $\theta_{IMU}$ and $\theta_{MAL}$ for vision impaired subjects.

The same experiment was conducted with the involvement of six male and four female vision impaired subjects. With a total of 138 trials, the mean correlation between the thigh angle $\theta_{IMU}$ and $\theta_{MAL}$ was 99.16% with a standard deviation of 0.67%. The minimum and maximum correlations were 96.38% and 99.92%. The mean RMSE for all 138 trials was 1.9317° with a standard deviation of 0.4949°. The distributions of correlation and RMSE between $\theta_{IMU}$ and $\theta_{MAL}$ for vision impaired subjects are shown in Figure 9. These results imply that the algorithm also gives comparable results in the case of vision impaired subjects.

### 3.2. Performance Evaluation

To assess the performance of GIOF, the number of calculations is compared against that of the complementary filter, which is known to be a faster filter that consume lower amount of computational resources. The first order complementary filter discussed in [14] was considered for this, which is given by

(3) $$\theta_{k}=\alpha \left(\theta_{k-1}+\omega_{k}\cdot \Delta t\right)+\left(1-\alpha \right)a_{k}$$

where $\theta_{k}$ and $\theta_{k-1}$ are the new and previous estimates of the thigh angle, $\omega_{k}$ is the current gyro reading, Δt is the sample time, and $a_{k}$ is the angle estimated by taking atan2 of the accelerometer readings. α is the filter coefficient which determines the composition of two estimations: angles estimated using gyroscope and by accelerometer, and 0≤α≤1.

The computation in (3) consists of four two-input floating-point multiplications and three two-input floating-point addition/subtraction operations. The acceleration and gyro tests of GIOF consist of three two-input floating-point multiplications, two two-input floating-point additions, two floating-point comparisons, and one logic operation. These two computations may be assumed to have comparable execution times. However, arctan consumes a number of floating-point calculations and may be considered as highly computationally expensive on low-end 8-bit microcontrollers having no floating-point processor. Therefore, performing arctan in each iteration requires long processor times. However, GIOF executes arctan only when conditions in (1) and (2) are satisfied, which makes the average execution performance of GIOF better than that of complementary filter on low-end 8-bit microcontroller platforms. The number of floating-point calculations required for a single iteration with Kalman filter is even higher because the parameter α in (3) is derived per each iteration [14].

The execution time for GIOF and complementary filter implementation were measured separately by implementing both in an Arduino Pro Mini, which incorporates an Atmel Atmega 328 (8-bit) microcontroller running at 8 MHz. Data were read from an MPU-9150 IMU. It was observed, in the MATLAB analysis, that the complementary filter gives correlation close to correlations mentioned in Section 3.1, when α=0.9999. Therefore, this value was used in the implementation.

The average execution time reported for the complementary filter implementation was approximately 570 μs and, for GIOF it was ~225 μs when not performing arctan and ~500 μs when performing arctan. The average execution time is ~250 μs for walking trials. This implies that the GIOF is computationally economical (by a factor of ½) compared to complementary filter. Further, although the complementary filter had similar correlations when α=0.9999 in MATLAB simulations, in real-time implementation, the output of complementary filter drifted for that α value. Therefore, α was selected as 0.999 for the implementation to avoid drifting. The drifting indicates that more contribution from $a_{k}$ is expected to avoid the drift. This makes the output be less smooth.

**Figure 10:**
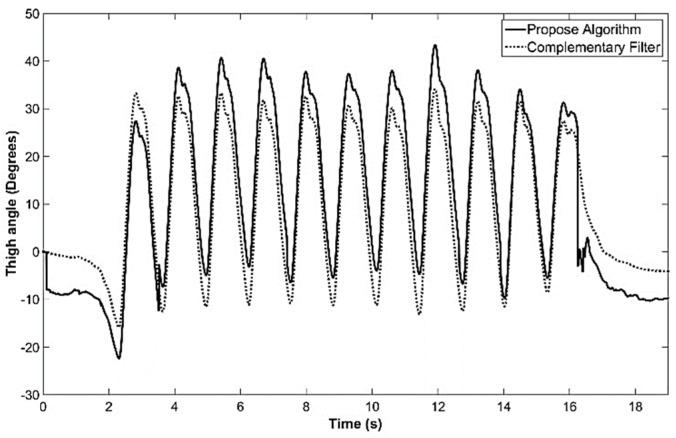
Thigh angle estimated in real-time in the IMU by GIOF and complementary filter captured for a trial when walking on a corridor.

All six (accelerometer and gyro) raw sensor data were low-pass-filtered with a second order Butterworth low-pass filter with cutoff frequency of 10 Hz. A gyro calibration was performed to estimate the offset of each gyro axis and the offset was subtracted from the raw sensor data during computation. The resulted thigh angle estimated by both GIOF and complementary filter in real-time in the microcontroller in a test trial captured while the subject was walking on a level corridor are shown in Figure 10.

## 4. Discussion

It was shown that the GIOF consumes one half of the computation time consumed by the complementary filter. The main reason for this is that the GIOF does not perform arctan in each computation. Instead, it performs arctan only when the accelerometer reading is stable. On the other hand, the complementary filter performs arctan in each iteration, hence consumes more processor time. Further, the complementary filter produces low drift when α is lower, but the output becomes less smooth. However, because the GIOF corrects the angle using the accelerometer, the drift in the output is avoided to a great extent.

It was observed that the GIOF performs comparably with both non-vision impaired and vision impaired subjects. This implies that GIOF can be used to estimate flexion and extension of vision impaired and non-vision impaired subjects during level walking. Compared with the RMSE of 3° reported in [4] for the forearm with Kalman filters, the RMSE of less than 2.5° reported by GIOF is accurate enough for thigh angle estimation for gait analysis. The GIOF has achieved better processing speed without compromising the accuracy.

Although memory-expensive, the moving average filter has shown better performance in removing the offset of the gyro reading. With properly selected window size, the drift in the estimated angle may be almost avoided with the moving average filter. However, the delay occurred in the filter becomes larger when the window width increases. For the window width selected in the analysis (150 samples), the delay occurred in the filter becomes 750 ms; this delay is not preferable for real-time implementations.

Although the GIOF was implemented to estimate pitch of the IMU, it can easily be extended to 2D and estimate both pitch and roll. Further, it may be extended to fuse magnetometer and gyroscope to estimate the yaw as well.

The experiment was conducted using a custom-made IMU with a specially designed 3D printed enclosure to make sure that the reflective markers of the Vicon system can be attached on top of the IMU to make the comparison easy. It should be noted that the GIOF can be implemented in a smartphone embedded with an accelerometer and a gyroscope. Hence, GIOF may be used for gait recognition in navigation applications with a smartphone placed in the front trouser pocket as explained in [7,8].

### Using a Single Thigh-Mounted IMU for Gait Phase Detection

**Figure 11:**
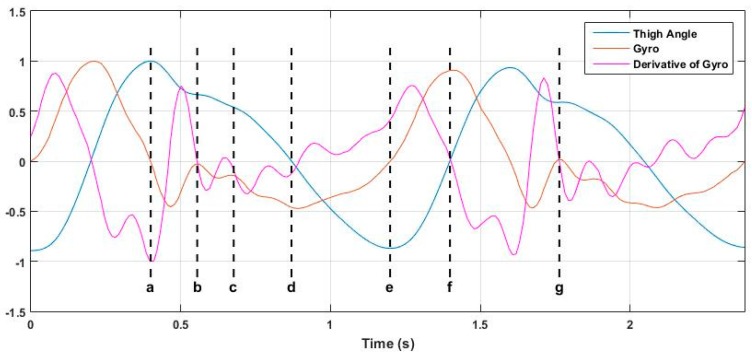
Normalized thigh angle, gyro signal, and time derivative of gyro signal.

It was observed that a single thigh-mounted IMU can be used to identify most of the phases of a stride cycle. The thigh angle estimated using GIOF, the gyro signal and the 1st derivative of the gyro signal is used in combination for this. Figure 11 shows the thigh angle, gyro signal and time derivative of gyro signal, all normalized to their peak value and drawn on the same time axis. The letters *a–g* represent the key points of the stride cycle identifiable in the thigh angle waveform. Although it is the positive peak of the thigh angle waveform, point ***a*** is not the initial contact as shown in Figure 2. Point ***a*** is the end of the swing of the leg. The initial contact point is point ***b*** of the diagram. Description of all phases identifiable in the thigh angle waveform is given in Table 2. It can be seen that except point ***c***, all other points can be detected by detecting zero crossings of the gyro signal or its time derivative. Table 3 shows which signal can be used to detect each point of the stride cycle. It should be noted that point ***g*** is as same as point ***b***. Further, the end of the loading response (point ***c***) may be approximately detected by the relative maxima of the time derivative of gyro signal.

It was observed that the three subphases of Swing phase (initial swing, mid swing, and terminal swing) cannot be distinguished in the thigh angle waveform. However, all five subphases of stance phase can be identified in the thigh angle waveform and four of these can be detected using zero crossing detectors on gyro signal and derivative of gyro signal. Although some gait phase detection techniques are discussed in literature (e.g., [15]), they involve force sensors to detect the foot contact on the floor. The above technique can be a significant achievement as it uses a single thigh-mounted IMU only.

## Figures and Tables

**Table 1:** Correlation statistics between $\theta_{MAL}$ and $\theta_{IMU}$.

	No. of Subjects	No. of Trials	Correlation Coefficient			
			Mean	Max	Min	Std
Males	10	196	0.9960	0.9995	0.9822	0.003114
Females	9	178	0.9955	0.9996	0.9733	0.003559
All	19	374	0.9958	0.9996	0.9733	0.003337

**Table 2:** Phases of gait cycle as identified in the thigh angle waveform.

Point/Points	Phase/Point of Gait Cycle
a	End of swing
b	Initial Contact
b–c	Loading Response
c–d	Mid Stance
d–e	Terminal Stance
e–f	Pre-Swing
f–g	Swing

**Table 3:** Signal feature that can be used to detect each point of the stride cycle.

Point	Signal to Detect Zero Crossing to Detect Each Point
a	Gyro
b	Derivative of gyro
c	-
d	Derivative of gyro
e	Gyro
f	Derivative of gyro
